# Utilizing Leadless Pacemakers in Extremely Elderly Patients With a Conventional Pacemaker System: A Two-Year Follow-Up Case Series Without Generator Extraction in High-Risk Scenarios

**DOI:** 10.7759/cureus.67003

**Published:** 2024-08-16

**Authors:** Man Fong Chu, Weng Chio Tam, Kuok Wun Lam, Chon Hou Chan, Màrio Évora, U Po Lam

**Affiliations:** 1 Cardiology, Centro Hospitalar Conde São Januário, Macau, MAC; 2 Dermatology, Centro Hospitalar Conde São Januário, Macau, MAC

**Keywords:** cardiac implantable electronic device, atrial fibrillation, extreme elderly, end of service, generator replacement, leadless pacemaker

## Abstract

Background and objectives

Leadless pacemakers, known for their safer clinical profile, offer significant advantages for elderly patients at a higher risk of complications associated with transvenous pacemaker procedures, particularly those susceptible to high-risk bleeding and infections related to cardiac implantable electronic device interventions. This study explores an alternative use of leadless pacemakers without removing existing transvenous systems, deviating from conventional generator replacement and lead re-interventions.

Methods

This study was conducted with full approval from the Institutional Review Board, Medical Ethical Committee, Centro Hospitalar Conde São Januário, Macau. Between January 2018 and December 2021, we conducted a retrospective case series involving extremely elderly individuals (aged 85 years or older) at a high risk of complications, necessitating either generator replacement or lead re-implantation. The study considered implanting a leadless pacemaker (Micra; Medtronic, Minneapolis, MN, USA) without removing the transvenous generator. For the primary endpoints, we evaluated procedure-related complications and clinical outcomes during hospitalization. Secondary endpoints included the stability of parameters and any unexpected interference or interactions between the two systems during the two-year follow-up.

Results

Eleven patients (aged 86-101) were enrolled, most receiving antiplatelet or anticoagulation therapy. Leadless pacemaker implantation proceeded without major complications or adverse clinical outcomes during hospitalization. Regular follow-up was conducted every three to six months for adjusting pacemaker parameters and interrogating each patient. Over two years, three patients died from non-cardiac causes: two from infection and one from spontaneous intracranial hemorrhage, while eight completed regular follow-ups. We didn’t detect any episodes of ventricular arrhythmias or intracardiac capture from the transvenous pacemaker system. We observed the stability in both the longevity and the voltage of the conventional generator battery, maintaining similar parameters without significant depletion (mean voltage decline: -0.07V/year). Parameters of the leadless pacemaker remained consistently normal without interference with existing pacing systems.

Conclusion

Implanting leadless pacemakers without removing transvenous pacemaker generators appears safe and effective for extremely elderly patients who are at high risk of complications. Comprehensive two-year follow-up supports the safety and viability of this approach. Opting for this approach instead of conventional generator replacement, with or without additional lead implantation, may be reasonable in this population. However, further research within this patient cohort, such as exploring long-term outcomes beyond two years or comparing clinical outcomes with conventional strategies, may be necessary.

## Introduction

Due to an increasingly aged population and extended life expectancies, the prevalence of pacemaker implantation procedures is rising, with approximately one in 50 among people aged 75 or older [1]. However, conventional pacemaker strategies entail unpredictable complications, such as pneumothorax, hemothorax, pocket hematoma, infections related to pacemaker systems, and lead-related issues (such as lead fracture and insulation break), with an overall estimated rate of approximately 6-10% across medical centers and operator expertise [2-4]. To address these concerns, leadless pacemakers (LP) have emerged as a safer alternative with more favorable clinical outcomes, mitigating complications associated with conventional systems [5,6]. Specifically, the LP system offers promise for elderly patients at a heightened risk of complications, including hematoma, venous disorders, and cardiac implantable electronic device (CIED) infections [7].

There are two types of LP, Micra VR/AV by Medtronic (Minneapolis, MN, USA) and AVEIR by Abbott (Abbott Park, IL, USA) [8]. The Micra system resides entirely within the right ventricle, with recommendations for a new implantation upon battery depletion to avoid myocardial tissue injury. It can be manually programmed off and is automatically deactivated at the end of its service life. According to existing knowledge, it is established that a single patient's right ventricle has the capacity to accommodate up to three Micra pacing systems throughout their lifetime, making it a reasonable choice for elderly patients requiring intracardiac pacing [9].

Generator replacement procedures present a unique set of potential complications, including pocket hematomas and CIED infections [10]. The overall incidence of CIED infections ranges from 1-4%, varying based on healthcare facilities (including procedural protocol, sterile environment and procedural time) and patient comorbidities [11]. Elderly individuals, particularly those predisposed to bleeding, face an escalated risk of pocket hematoma and CIED infections [12].

CIED infections can lead to adverse clinical outcomes, particularly in the elderly. Specific risk factors beyond bleeding susceptibility and pocket hematoma include chronic kidney disease, diabetes mellitus, prolonged steroid usage, lung disease, cerebrovascular ailments, previous heart surgeries, extended procedure times, temporary catheter presence, and re-intervention procedures [11]. Generator replacement, akin to system upgrades and lead revisions, is considered a revision or re-intervention procedure [12]. This heightened risk was evidenced by a notably higher rate of CIED infections following generator replacement when compared to new implantations [13]. In summary, elderly patients with multiple comorbidities undergoing generator replacement as a revision procedure present a higher risk of CIED complications compared to new implantation. LP may be a reasonable alternative to conventional generator replacement [14].

LPs offer an innovative approach by eliminating the need for transvenous leads and subcutaneous pockets, potentially reducing hardware contamination during bacteremia [5]. The leadless system's encapsulation by endothelium serves to diminish bloodstream infections, as the encapsulated device can be sealed off from bacteria. Additionally, turbulent blood flow within the right ventricle discourages bacterial adherence compared to static blood flow within the superior vena cava [15]. Furthermore, a recent meta-analysis showed that LPs had lower rates of CIED infections [16]. LPs are considered a viable alternative option for very elderly patients at risk of CIED infections.

The risk-benefit assessment of leadless pacing systems compared to conventional ones should be conducted on an individual basis. Periprocedural complications of LP implantation include dislodgement, vascular injury, elevated pacing thresholds, and cardiac perforation, with the latter being more prevalent in female patients with a low body mass index and smaller hearts [17,18]. 

Notably, while a case report highlighted the concurrent utilization of LPs in very elderly patients with abandoned transvenous pacemaker (TP) systems, comprehensive case series are needed to validate the efficacy and safety of LPs in patients with depleted TP batteries [14]. The utilization of LP, without the removal of transvenous generator, as an alternative to traditional generator replacement in extremely elderly patients remains a plausible therapeutic option. However, the current evidence remains inconclusive.

This study aims to explore LP implantation as an alternative for generator replacement or pacemaker lead reimplantation in extremely elderly patients without transvenous generator removal, focusing on those at high risk and susceptibility to TP system complications. A comprehensive two-year patient follow-up was conducted to assess the safety and effectiveness of this approach, including meticulous modifications to LP and unextracted TP system parameters.

## Materials and methods

Patient selection

This study was conducted with full approval from the Institutional Review Board, the Centro Hospitalar Conde de São Januário Hospital Medical Ethical Committee. It involved a retrospective enrollment process at a single medical center between January 2018 and December 2021. The study targeted elderly individuals aged 85 years or older, identified as candidates for either pacemaker generator replacement or re-implantation of a new pacemaker system due to battery depletion or lead disorders. These patients, apart from their age, presented two or more specific comorbidities significantly increasing their vulnerability to complications related to CIEDs, including factors such as the use of anticoagulation, chronic kidney disease, and poor glycemic control, among others.

While the primary options for these patients were either generator replacement or the implantation of new leads, the study also considered LP implantation as a viable alternative. This consideration was prompted by the increased risk of CIED infection and other complications associated with the traditional transvenous system, especially in elderly patients. We held informed discussions with both patients and their family members to obtain consent for the LP implantation procedure, explaining the risks and benefits of both conventional and alternative strategies.

We excluded patients with an initial End of Service (EOS) program for their transvenous system because their devices could not be interrogated. We also excluded patients who were reluctant to consider the alternative approach. After receiving approval from the patients, we implanted the LP, specifically, the Medtronic Micra model, without removing the existing transvenous system.

Procedure protocol, device interrogation and two-year follow-up

Regarding the procedural protocol, before LP implantation, the pacing mode of the existing transvenous system was transitioned to VVI (ventricle paced, ventricle sensed, and pacemaker inhibited in response to a sensed beat) mode with a lowest rate of 30 or 45, and the threshold was set to the minimum values, except in cases where the patient was entirely pacemaker-dependent. This step was taken to ensure that the ventricle was not captured under the lowest threshold. 

We also recorded technical specifications, such as model numbers, manufacturers, and key features of both of LP and TP systems. After implanting the LP, the mode of the old transvenous system was permanently switched to VVI mode with a lowest rate of 30-45 beats per minute and the lowest threshold settings (0.3-0.5mv/0.03-0.4ms) mode, or ODO mode. If the specific pacemaker model, such as the Abbott Endurity, permitted it, the pacing mode of the TP was deactivated. Simultaneously, thorough testing and interrogation of the LP were conducted to identify any inappropriate sensing interactions between the new LP and the old TP. 

Following the implantation, the study conducted a two-year follow-up involving periodic interrogations of both the TP and LP. Regular follow-up was also conducted every three to six months for adjusting pacemaker parameters and interrogating each patient. 

Procedural technique, medical team and data interpretation 

All operators performing LP implantation should be experienced and have completed a professional training program for the procedure. Operators should perform at least 20 LP implantations annually to be considered experienced. At least two cardiologists were involved for each procedure. With the support of fluoroscopy and contrast agents, we performed the LP implantation. 

During follow-up, all parameters were interpreted, analyzed and confirmed by two cardiologists at the pacemaker clinics. This approach helps ensure the accuracy of data interpretation.

Primary and secondary endpoints to evaluate the safety and effectiveness

For the primary endpoints, we evaluated clinical measures and outcomes during hospitalization, including procedure-related complications, duration of hospital stay, cardiovascular events, cerebrovascular events, all-cause death, and complications related to the transvenous pocket. Secondary outcomes included parameter stability, unexpected pacing-related ventricular arrhythmias, and any unexpected interference or interactions between the two systems during the two-year follow-up.

## Results

In this case series study, as detailed in Table 1, a total of 11 patients, aged between 86 and 101, were enrolled. Nine of these patients were recommended for transvenous generator replacement, while two patients, who presented right ventricle lead dysfunction, were advised to undergo re-implantation of transvenous leads. The majority of our patient cohort had underlying atrial fibrillation or coronary artery disease and were receiving anticoagulant or antiplatelet therapy. In consideration of specific concerns related to a heightened bleeding risk and increased susceptibility to complications associated with CIEDs, we opted for LP implantation (specifically Micra; Medtronic) without removing the old transvenous system. 

**Table 1:** Baseline characteristics of enrolled patients SD: standard deviation

	Leadless pacemaker implantation (N=11)
Age (mean, years old) +/-SD	92.2 +/-5.2
Asia	100%, (11)
Body mass index (mean, kg/m2) +/-SD	23.6 +/-3.2
Female	73%, (8)
Procedure indication	
Battery depletion	82%, (9)
Lead failure	18%, (2)
Atrial fibrillation	73%, (8)
Current anticoagulation/antiplatelet	91%, (10)
Chronic kidney disease	100%, (11)
Diabetes mellitus	18%, (2)
Previous cardiac surgery	0%, (0)
Mean procedure time min +/-SD	28.8 +/-7.6
Local anesthesia	100%, (11)

The primary outcomes are summarized in Table 2. The procedures proceeded without major complications such as cardiac perforation, lead dislodgement, or pacemaker-related arrhythmias. However, one patient encountered a vascular injury due to vessel tortuosity and challenging puncture. With appropriate and timely management, this patient experienced a full recovery. None of our patients suffered from transvenous pocket complications, cardiovascular events, cerebrovascular events, or all-cause death during hospitalization. All patients were discharged within five days, except for one who required additional time for the investigation of an incidental intra-abdominal mass.

**Table 2:** The primary endpoint focused on clinical outcomes during hospitalization

Procedure-related complication (N=11)	
Pericardial effusion (number)	0
Femoral wound infection (number)	0
Any Lead dislodgement (number)	0
Pacemaker related Ventricular arrhythmia (number)	0
Femoral vein thrombosis (number)	0
Pacemaker syndrome (number)	0
Vascular injury (number)	1
Procedure-related death (number)	0
In-hospital clinical outcome (N=11)	
Hospital stay > 5 days (number)	1
Cardiovascular event (number)	0
Cerebrovascular event (number)	0
All-cause death (number)	0
Transvenous pocket complication (number)	0

**Figure 1:**
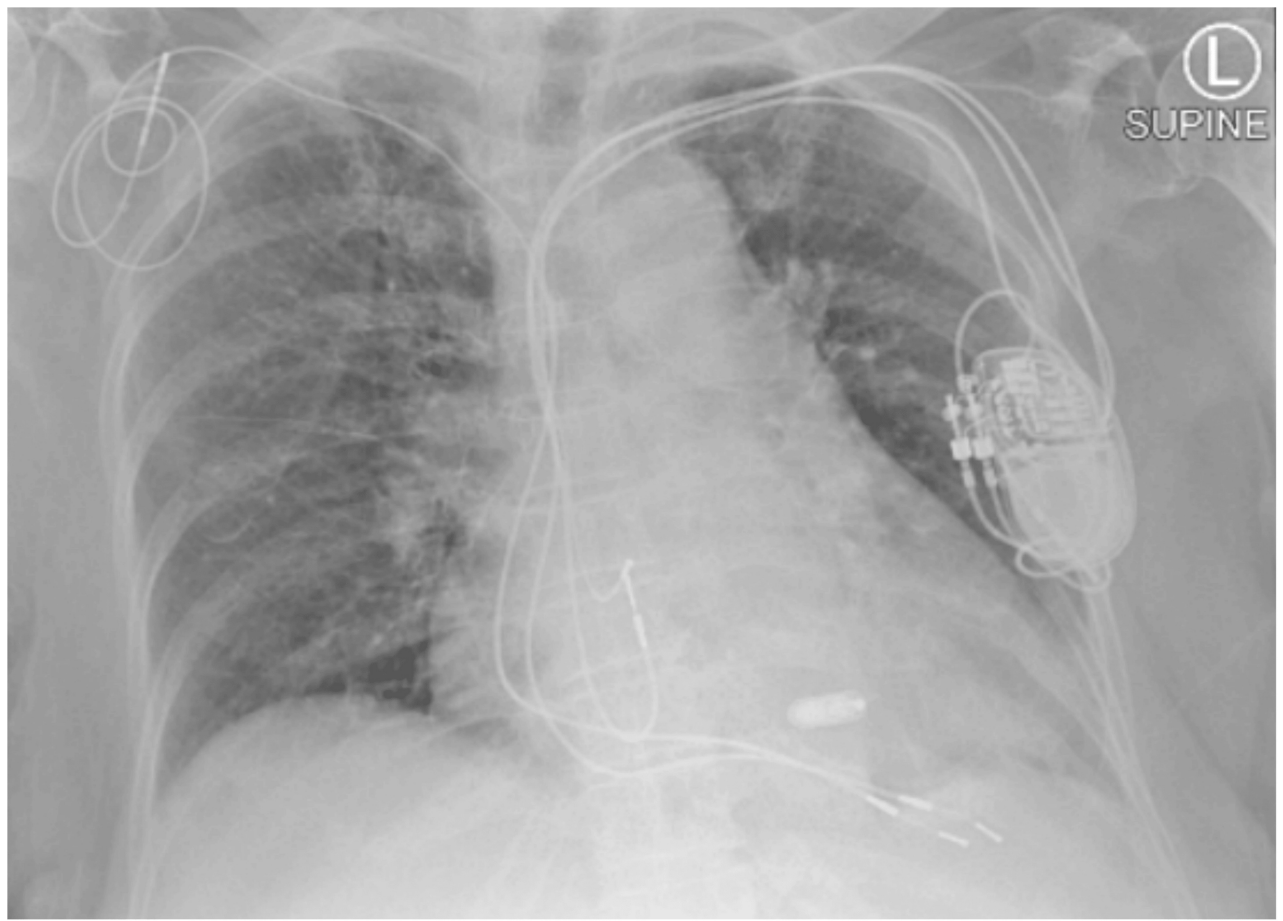
Chest X-ray after leadless pacemaker implantation Perform a chest X-ray to assess the positioning of both leadless and traditional pacemaker systems. Confirm the placement of the leadless pacemaker by verifying its location in the appropriate septal region and ensuring there is no dislodgement of transvenous leads after implantation.

**Figure 2:**
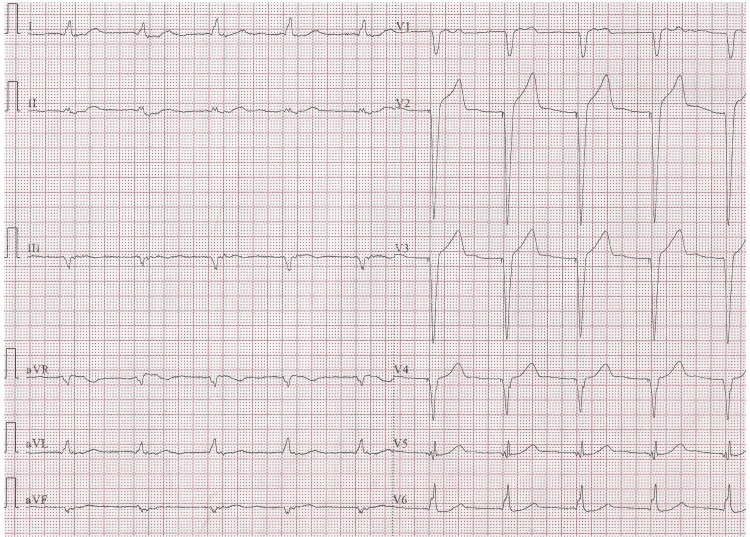
12-lead electrocardiogram after leadless pacemaker implantation Perform an electrocardiogram to confirm the proper pacing and sensing functions of the leadless pacemaker set to VVI (ventricle paced, ventricle sensed, and pacemaker inhibited in response to a sensed beat) mode at 60 beats per minute. Ensure that there is no unexpected erratic pacing from the transvenous pacemaker system.

Table 3 provides an overview of the major comorbidities associated with an increased risk of complications from TP interventions and the status of the existing TP systems. Table 4 presents the parameters of the LP post-implantation, all of which were within normal ranges. It also includes information about the pre-existing TP model and the adjustments made to TP parameters during Micra implantation. Notably, no captured rhythm originated from the TP under minimal pulse width and threshold settings. Following LP implantation, we conducted a chest X-ray to assess the positioning of both pacemakers and obtained a 12-lead ECG to confirm the appropriate sensing and pacing function of the pacemaker systems, as illustrated in Figure 1 and Figure 2.

**Table 3:** Overview of the status of existing transvenous pacemaker and major comorbidities associated with an increased risk of complications from transvenous pacemaker interventions Af: atrial fibrillation, AV: atrioventricular, NOAC: novel oral anticoagulant, CAD: coronary artery disease, HF: heart failure, DM: diabetes mellitus, CKD: chronic kidney disease, TP: transvenous pacemaker, RV: right ventricle

Patient No	Age	gender	Indication of existing TP implantation	Status of TP system	Number of concerned Comorbidities	Major concerned comorbidities associated with complications of conventional approach	
#1	90 y/o	F	high degree AV block	Longevity<12 months	2	Bowen’s disease generalized skin erosion, CKD	Asthenic with possible wound dis-adherence, easy bleeding from skin erosion
#2	93 y/o	F	Af with long pause	Longevity<6 months	3	Af, Heart Failure, CKD	Anticoagulation with high bleeding risk
#3	95 y/o	M	Af with long pause	Longevity: 5 months, RV lead high threshold (5V/1ms)	3	Af, Dementia, CKD	Anticoagulation with high bleeding risk
#4	86y/o	F	Af with long pause	RV lead failure (lead impedence: 140 ohms)	3	Af, DM without control, CKD	Anticoagulation with high bleeding risk, poor glycemic control
#5	86y/o	M	Af with long pause	RV lead fracture (lead impedence>2000 ohms)	2	Af, CKD	Anticoagulation with high bleeding risk, poor glycemic control
#6	100y/o	F	Sick sinus syndrome	Longevity< 1 year	4	Af, Dementia, CAD, CKD	Current antiplatelet use with high bleeding risk
#7	101y/o	M	Complete AV block	Longevity< 6 months, high threshold of RV lead	3	Af, Dementia, CKD	Anticoagulation with high bleeding risk
#8	91y/o	F	Complete AV block	Longevity: 7 months, RA lead dysfunction	2	Af, CKD	Anticoagulation with high bleeding risk
#9	89y/o	F	Sick sinus syndrome	Longevity<1 month	3	Af, Parkinson disease, CKD	Anticoagulation with high bleeding risk
#10	91 y/o	F	Sick sinus syndrome	Longevity: 3 months	2	CAD, CKD	Current antiplatelet use with high bleeding risk
#11	87y/o	F	Sick sinus syndrome	Longevity: 4 months	3	CAD, Dementia, CKD	Current antiplatelet use with high bleeding risk, poor self-care

**Table 4:** Parameters of the leadless pacemaker (regular font), model of the pre-existing transvenous pacemaker and the adjustments made to the transvenous pacemaker (italic font) during the implantation of the leadless pacemaker BPM: beat per minute, VVI: ventricle paced, ventricle sensed, and pacemaker inhibited in response to a sensed beat, ODO: no pacing, atrial and ventricular sensing, no action

Patient No	Age	gender	Parameters of Leadless pacemaker system (MICRA)				Conventional transvenous pacemaker (Model)	Parameters modification of transvenous pacemaker			
			Sensing (mV)	threshold (V/ms)	Impedence (Ohms)	Lower rate (BPM)		Mode	lower rate (BPM)	Minimal Amplitude/pulse width (V/ms)	Capture during minimal threshold
#1	90 y/o	F	9.2	0.38/0.24	580	60	Medtronic SIGMA SDR 303	VVI	30	0.5/0.03	no
#2	93 y/o	F	9.5	0.88/0.24	620	60	Medtronic SIGMA SDR 303	VVI	30	0.5/0.03	no
#3	95 y/o	M	11	0.38/0.24	790	70	Medtronic, Relia RESR01	VVI	30	0.5/0.12	no
#4	86y/o	F	>20	0.38/0.24	690	55	Abbott, Endurity 1162	Pacing deactivated	-	nil	nil
#5	86y/o	M	6	0.63/0.24	720	60	Abbott, Regency SC	VVI	45	0.3/0.43	no
#6	100y/o	F	11	0.5/0.24	1350	55	Medtronic, Relia RESR 01	VVI	30	0.5/0.12	no
#7	101y/o	M	8	0.38/0.24	1400	60	Medtronic, Relia, RESR01	VVI	30	0.5/0.12	no
#8	91y/o	F	5	0.38/0.24	740	55	Medtronic Advisa DR MRI A3DR01	ODO	-	nil	nil
#9	89y/o	F	9.6	1.13/0.24	710	70	Medtronic, Adapta L ADDRL1	VVI	30	0.5/0.12	no
#10	91 y/o	F	6.6	0.63/0.24	1370	60	Medtronic, Advisa DR MRI A3DR01	ODO	-	nil	nil
#11	87y/o	F	10	0.5/0.24	750	60	Medtronic Advisa DR MRI A3DR01	ODO	-	nil	nil

Patients underwent regular follow-ups at the pacemaker clinic, involving thorough interrogations of both the newly implanted leadless system and the existing transvenous system. The secondary outcomes are detailed in Table 5 (12 months) and Table 6 (24 months) respectively. Unfortunately, three patients passed away due to non-cardiac causes during the two-year follow-up, specifically pneumonia, intracranial hemorrhage, and retroperitoneal abscess (Table 6). However, it is noteworthy that eight patients successfully completed regular follow-ups during this duration. Remarkably, there were no recorded instances of ventricular pacing or ventricular arrhythmias via the interrogations of the TP. During each examination, overdrive pacing (VOO) was executed via the old transvenous system, employing minimal threshold settings to ensure the absence of intracardiac capture from the TP system. Furthermore, we verified that the Micra leadless system, set to VVI mode at 30 beats per minute, did not detect any pacing rhythm from the TP. Subsequently, a 12-lead electrocardiography was obtained during the test for further confirmation. Analysis of the histogram of the LP revealed the highest ventricular rate ranging from approximately 90 to 140 beats per minute. Importantly, we observed this stability in both the longevity of the TP system and the voltage of the TP battery, maintaining similar parameters without significant battery depletion due to minimal workload settings. The mean voltage decline of TP battery was approximately -0.07V per year. Concurrently, the parameters of the Micra in all patients remained within the normal range during follow-up, with no instances of interference or interaction between the two pacing systems.

**Table 5:** After 12 months following Micra (Medtronic, Minneapolis, MN, USA) implantation: regular follow-ups, adjustments, and interrogations of both leadless (regular font) and existing transvenous (italic font) systems at the pacemaker clinic This table summarizes the secondary outcomes related to the pacemaker parameters of both systems over 12 months. We did not record any instances of ventricular pacing or ventricular arrhythmias during interrogations of the transvenous system. The histogram analysis of the leadless pacemaker shows that the highest ventricular rates ranged from approximately 90 to 140 beats per minute. NA: high ventricular rate and pacing percentage monitor was terminated after elective replacement indicator status in some specific model, BPM: beat per minute, VVI: ventricle paced, ventricle sensed, and pacemaker inhibited in response to a sensed beat, ODO: no pacing, atrial and ventricular sensing, no action, EGM: intracardiac electrogram

Patient No	Age	gender	Parameters of leadless pacemaker (12 months after Micra implantation)						Parameters of conventional transvenous pacemaker (12) months after Micra implantation)						
			Sensing (mV)	threshold (V/ms)	Impedance (Ohms)	Sensed EGM from transvenous pacing capture	Lower rate (BPM)	Highest histogram	Longevity	battery voltage	Mode	lower rate (BPM)	Pacing (%)	Ventricular arrhythmia detection	Capture during minimal threshold
#1	90 y/o	F	7.7	0.5/0.24	510	Nil	60	120	11 months	2.72V	VVI	30	0	no	no
#2	93 y/o	F	9.5	0.38/0.24	620	Nil	60	120	1 month	2.6V	VVI	30	0	no	no
#3	95 y/o	M	15	0.5/0.24	650	Nil	70	100	Replace pacer	2.74V	VVI	65	NA	NA	no
#4	86y/o	F	16	0.5/0.24	580	Nil	55	110	12 years	3.02V	Off	-	0	no	no
#5	86y/o	M	8	0.38/0.24	550	Nil	60	130	>1 year	2.78V	VVI	45	0	no	no
#6	100y/o	F	7.7	0.5/0.24	660	Nil	55	90	9 months	2.71V	VVI	30	0	no	no
#7	101y/o	M	10	0.38/0.24	480	Nil	60	120	6 months	2.56V	VVI	30	0	no	no
#8	91y/o	F	5	0.38/0.24	740	Nil	55	120	<6 months	2.8V	ODO	-	0	no	no
#9	89y/o	F	11	0.38/0.24	640	Nil	70	130	Replace pacer	2.58V	VVI	65	NA	NA	no
#10	91 y/o	F	10	0.5/0.24	560	Nil	60	100	3 months	2.85V	ODO	-	0	no	no
#11	87y/o	F	10	0.38/0.24	750	Nil	60	140	4 months	2.84V	ODO	-	0	no	no

**Table 6:** After 24 months following Micra (Medtronic, Minneapolis, MN, USA) implantation: regular follow-ups, adjustments, and interrogations of both leadless (regular font) and existing transvenous (italic font) systems at the pacemaker clinic This table summarizes the secondary outcomes related to the pacemaker parameters of both systems over 24 months. During the two-year follow-up, three patients passed away due to non-cardiac causes. We observed stable longevity and voltage in the transvenous pacemaker battery. We did not record any unexpected erratic pacing or ventricular arrhythmias during interrogations of the transvenous system. Concurrently, the parameters of the Micra remained within the normal range for all patients throughout the follow-up period. NA: high ventricular rate and pacing percentage monitor was terminated after elective replacement indicator status in some specific model, BPM: beat per minute, VVI: ventricle paced, ventricle sensed, and pacemaker inhibited in response to a sensed beat, ODO: no pacing, atrial and ventricular sensing, no action, EGM: intracardiac electrogram

Patient No	Age	gender	Parameters of leadless pacemaker (24 months after Micra implantation)						Parameters of conventional transvenous pacemaker (24 months after Micra implantation)							
			Sensing (mV)	threshold (V/ms)	Impedance (Ohms)	Sensed EGM from transvenous pacing capture	Lower rate (BPM)	Highest histogram	Longevity	battery voltage	Battery voltage decay/year	Mode	lower rate (BPM)	Pacing (%)	Ventricular arrhythmia detection	Capture during minimal threshold
#1	90 y/o	F	7.7	0.5/0.24	510	Nil	70	120	Replace pacer	2.59V	-0.13V	VVI	65	NA	NA	no
#2	93 y/o	F	9.5	0.38/0.24	620	Nil	70	120	Replace pacer	2.3V	-0.3V	VVI	65	NA	NA	no
#3	95 y/o	M	15	0.5/0.24	650	Nil	70	100	Replace pacer	2.73V	-0.01V	VVI	65	NA	NA	no
#4	86y/o	F	16	0.5/0.24	580	Nil	55	110	10 years	3.02V	0V	Off	-	0	no	no
#5	86y/o	M	8	0.38/0.24	550	Nil	60	130	>1 year	2.78V	0V	VVI	45	0	no	no
#6	100y/o	F		Passed away due to pneumonia, 02/2023, 21st month												
#7	101y/o	M	10	0.38/0.24	480	Nil	60	120	<1 month	2.52V	-0.04V	VVI	30	0	no	no
#8	91y/o	F	5	0.63/0.24	530	Nil	55	130	6 months	2.8V	-0.02V	ODO	-	0	no	no
#9	89y/o	F		Passed away due to intracranial hemorrhage, 04/2023, 16th month												
#10	91 y/o	F		Passed away due to retroperitoneal infection, 05/2023, 17th month												
#11	87y/o	F	9	0.5/0.24	680	Nil	60	130	Replace device	2.8V	-0.04V	ODO	-	0	no	no

Upon the termination of the follow-up period, we identified the elective replacement indicator (ERI) status in Patients #1, #2, and #3 (Table 5). In these three patients, the mode of the TP automatically switched to VVI at a lower rate of 65, employing minimal threshold and pulse field settings specific to their TP model (Medtronic). To prevent unnecessary pacing from the TP system, we adjusted the lower rate of the LP to 70 beats per minute. Under the ERI status of TP, the high ventricular rate monitor automatically terminated to minimize battery consumption, and the ventricular arrhythmia monitoring in the TP system deactivated. This prompted a shift in focus to the evaluation of the highest histogram from the LP, consistently falling within the range of 100-120. Moreover, we methodically confirmed the absence of ventricular capture under the ERI settings of the TP system. 

## Discussion

Advanced age and susceptibility to bleeding are associated with an increased risk of CIED infection [12,19]. Given the growing life expectancy and the need to minimize the risk of CIED infection, the utilization of LPs has emerged as a viable solution to reduce complications related to TP implantation. To our knowledge, this study marks the first case series evaluating the use of LP in conjunction with an existing TP system. Our findings illustrate that implanting an LP (N=11), without removing abandoned TP generators, could present a reasonable alternative for advanced-age patients and those at high risk of complications (i.e., more than two comorbidities associated with CIED complications), who previously underwent conventional TP implantations and now require battery replacement or additional lead implantation. Our primary endpoint demonstrates favorable clinical outcomes without major complications during hospitalization. Over a two-year follow-up period, the secondary endpoint shows stable parameters and the absence of unexpected interference or interactions between the two systems. We have also established the safety and efficacy of LPs as an alternative treatment modality. Although this approach remains controversial according to current guidelines, our findings offer valuable insights into the feasibility and advantages of using LPs for this specific patient population.

In our study, some patients encountered issues related to lead dysfunction and lead failure within the traditional TP system. While laser lead extraction is a potential solution, it presents a high risk of complications due to the extended duration that the old transvenous leads have been in place. Furthermore, lead extraction is a technically demanding procedure and may pose an elevated risk of major peri-operative complications, including cardiac perforation, incomplete lead extraction and tear of the superior vena cava, particularly in the case of extremely elderly patients [20,21]. In such circumstances, another approach is to implant a new TP system without attempting to extract the failed leads. However, the addition of more leads via the superior vena cava in a subsequent pacemaker implantation procedure could elevate the risk of superior vena cava obstruction [22]. Moreover, the presence of multiple leads entering the right ventricle can lead to significant tricuspid regurgitation, resulting in clinical symptoms of heart failure [23,24]. Due to the above concern, the implantation of an LP offers a viable solution for extremely elderly patients in these situations. This approach is characterized by shorter procedure time and the simultaneous resolution of lead failures and comorbidities, thus mitigating the risks associated with another TP implantation and lead extraction.

All our patients received the Micra VR system implantation, primarily because the majority of our patients either had underlying atrial fibrillation rhythms or experienced limited mobility with mild to moderate fragility. As far as we know, the Micra VR system has the potential to induce atrioventricular dyssynchronization, which can lead to pacemaker syndrome, marked by clinical symptoms like palpitations and heart failure. In MARVEL 2 study, in cases where atrial sensing was indicated or the potential for atrioventricular dyssynchronization exists, the Micra AV LP was recommended to mitigate the risk of pacemaker syndrome [25]. One report has demonstrated the feasibility and safety of upgrading from a VVI intracardiac LP (Micra VR) to an atrioventricular synchronous LP (Micra AV) in a 72-year-old patient who initially opted for Micra VR due to pacemaker syndrome. Notably, this upgrade resulted in no complications or adverse events related to the simultaneous presence of two LP systems in the right ventricle [26]. Fortunately. none of our patients developed pacemaker syndrome or experienced palpitations following LP implantation in our study.

In this case series study, the majority of TPs required generator replacement due to battery depletion. It's worth noting that we did not include any patients with the initial EOS program from the TP system in our study. Nevertheless, in situations involving extremely elderly patients at a high risk of complications, we pursued an alternative approach by opting for the implantation of the Micra LP instead of traditional generator replacement. For certain models of the TPs (such as the Abbott Endurity), pacing mode can be deactivated or switched off. In instances where deactivation or switching off is not possible, we modified the traditional pacemaker's mode to VVI with the lowest rate (30-45/min), the lowest threshold (0.3-0.5V), and the shortest pulse width (0.03-0.12ms), or ODO mode. This adjustment was made to prevent unnecessary pacing from the old transvenous system and to avoid unexpected interference between the simultaneous presence of two pacemaker systems.

It is noteworthy that the EOS program may engender unanticipated pacing rhythm as it predominantly prioritizes ventricular pacing [27]. Unfortunately, the device telemetry is unavailable for interrogation during the EOS status. The specific mode of pacemaker during the EOS program varies depending on the manufacturer (e.g., Boston: VVI at 50 beats per minute, Medtronic: VVI at 65 beats per minute, and Abbott: VVI at 67.5 beats per minute), all with unstable sensing thresholds [28]. Erratic pacing during the end-of-service status may lead to ventricular arrhythmias, potentially requiring the removal of the old generator. Interestingly, we observed that ERI status can persist with similar parameters for a prolonged period after modifying parameters to their minimal and lowest settings in our study. The longevity of the un-explanted old generator showed no significant difference. During the two-year follow-up, we did not observe any significant ventricular arrhythmias induced by either the leadless or traditional pacemakers, and there were no reports of erratic pacing from the pre-existing TP during pacemaker clinic check-ups. 

Even though the batteries of the TP systems did not reach the EOS status during follow-up, it is essential to routinely assess the TP system to confirm whether its EOS program has been activated. In our study, spanning over a period of two years, we observed that the TP system of Patient #2 transitioned to the EOS mode at 48 months after LP implantation. Following the manufacturer's EOS settings (Medtronic Sigma SDR 303), the mode was automatically changed to VVI 65 beats per minute with unstable sensing and pacing output. Notably, during the 48-month follow-up of Patient #2, we did not observe any erratic pacing from the abandoned transvenous system. The LP parameters at VVI mode 70 beats per minute remained within normal ranges, with no instances of high ventricular rates recorded in the histogram, nor any unexpected interferences arising from the simultaneous presence of two pacemaker systems. In the event that the EOS program triggers unexpected pacing, potentially leading to lethal arrhythmias, the removal of the abandoned TP generator may be deemed necessary.

It is important to note that not all individuals are suitable candidates for LP implantation. While LPs offer a less invasive and generally safer alternative, they do have their disadvantages and limitations. In the LEADLESS II trial, major complications were estimated to occur in 6-7% of cases, including dislodgement (1.1%), vascular injury (1.2%), elevated pacing thresholds (0.8%), and cardiac perforation (1.5%) [17]. Cardiac perforation is more common in female patients with a low body mass index and smaller hearts [18]. To reduce the risk of cardiac perforation, it is advisable to position the LP securely within the right ventricular septal wall region. Additionally, tortuous venous vessel and severe scoliosis can complicate LP implantation due to technical difficulties. In our study, we encountered one case of vascular injury during the procedure, which was promptly managed. Moreover, compared to TP systems, LPs have limited programming options. Therefore, careful patient selection prior to LP implantation is essential to minimize the risk of periprocedural complications.

The majority of our cases presented with underlying conditions, such as atrial fibrillation and coronary artery disease, for which anticoagulation or antiplatelet medications were prescribed, making them susceptible to bleeding. While interrupting these medications might be considered in cases of pocket complications arising from TP interventions, persistent interruption could increase the risk of cerebrovascular stroke or cardiovascular events [29,30]. Our patients stopped anticoagulation for only one day before the procedure, without prolonged interruption. Our therapeutic approach, involving LPs in these scenarios, may inadvertently overlook this concern. 

Limitation

This case series study has several limitations. Firstly, the relatively small sample size of 11 patients may limit the generalizability of the findings and impact the robustness of our conclusions. To address potential biases, we employed standardized data collection and analysis protocols; however, the retrospective nature of the study and the lack of a control group may still introduce selection and information biases. 

Additionally, all patients in this study were extremely elderly individuals of Asian descent, which may restrict the applicability of the results to other ethnic groups. Future studies should include a more diverse population.

The study also lacks long-term data beyond the two-year follow-up period, limiting our insights into the extended outcomes. Future research should extend follow-up durations to three to four years and include specific long-term cardiovascular endpoints. Moreover, this study did not address cost-effectiveness considerations related to LP implantation compared to traditional strategies. Future research should incorporate economic evaluations to provide a comprehensive assessment of the cost-benefit ratio of LPs versus TPs.

## Conclusions

In conclusion, this case series demonstrates that using LPs without removing TP generators is both feasible and effective for extremely elderly patients at high risk of complications. Key outcomes include the absence of major complications and adverse clinical events during hospitalization, along with stable parameters over a two-year follow-up. These results underscore the safety and effectiveness of this approach. By avoiding the need to extract existing TP generators, this method deviates from conventional practices of generator replacement and additional lead implantation, thereby reducing complications associated with TP systems.

Future research should focus on larger, multi-center studies with extended follow-up and diverse patient populations to validate these findings and evaluate the broader applicability of this approach.
